# Relief of Pain by the Outward Application of Hydrate of Chloral

**Published:** 1877-06

**Authors:** 


RELIEF OF PAIN BY THE OUTWARD APPLICATION OF HYDRATE OF CHLORAL.—Dr. W B. Kesteven (*Lancet*, Feb. 10, 1877) calls attention to the benefit to be derived in the relief of pain by the external application of the hydrate of chloral. for the knowledge of which he is indebted to his friend Dr. T. S. Dowse, of the Central London Sick Asylum, Highgate. The vast field that is afforded by these metropolitan institutions for the cultivation of practical medicine is not neglected by Dr. Dowse, but is turned by him to good account. The results of the trial of hydrate of chloral as an external application have lately been given. Dr. Kesteven adds his experience of this mode of using this drug, in order that it may become widely kuown.

He has tried it with great success in neuralgic pains and in cancer of the breast, in cases in which other sedatives and narcotics have failed to give relief.

The mode of application is by the saturation of folds of lint of the size of the part to which it is to be used, brought into close contact, then covered with three or four layers of lint covered with oil silk or spongio-piline wrung out of hot water. The application to raw surfaces, of course, requires some care in manipulation.

The strength of the solution is about four drachms to sixteen ounces of water. The addition of a small quantity of glycerine is is advantageous. Chloride of zinc or perchloride of iron can be combined with the chloral in certain cases.

